# CSF in Epileptic Prodromal Alzheimer's Disease: No Diagnostic Contribution but a Pathophysiological One

**DOI:** 10.3389/fneur.2021.623777

**Published:** 2021-08-03

**Authors:** Benjamin Cretin, Olivier Bousiges, Geoffroy Hautecloque, Nathalie Philippi, Frederic Blanc, Laure Dibitonto, Catherine Martin-Hunyadi, François Sellal

**Affiliations:** ^1^Unité de Neuropsychologie, Service de Neurologie et Hôpital de jour de Gériatrie, pôle de Gériatrie, Hôpitaux Universitaires de Strasbourg, Strasbourg, France; ^2^Centre Mémoire, de Ressources et de Recherche d'Alsace, Strasbourg-Colmar, France; ^3^University of Strasbourg and CNRS, ICube laboratory UMR 7357 and FMTS (Fédération de Médecine Translationnelle de Strasbourg), Team IMIS/Neurocrypto, Strasbourg, France; ^4^Centre de Compétences des démences rares des Hôpitaux Universitaires de Strasbourg, Strasbourg, France; ^5^University Hospital of Strasbourg, Laboratory of Biochemistry and Molecular Biology, CNRS, Laboratoire de Neurosciences Cognitives et Adaptatives (LNCA), UMR7364, Strasbourg, France; ^6^Service de Neurologie, Hospices Civils de Colmar, Colmar, France; ^7^Unité INSERM U-1118, Faculté de Médecine de Strasbourg, Strasbourg, France

**Keywords:** Alzheimer's disease, late onset epilepsy, mild cognitive impairment, cerebrospinal fluid, small vessel disease

## Abstract

**Objective:** To study whether cerebrospinal fluid (CSF) analysis may serve as a diagnostic test for the screening of epilepsy in sporadic prodromal Alzheimer's disease (AD).

**Methods:** A total of 29 patients with epileptic prodromal sporadic AD patients (epADs) were included and were retrospectively compared with 38 non-epileptic prodromal AD patients (nepADs) for demographics, clinical features, Mini-Mental Status Examination (MMSE) results, CSF biomarkers, and electro-radiological features.

**Results:** Our study did not show any significant differences in CSF biomarkers regarding neurodegeneration, albumin levels, and inflammation between epADs and nepADs. The epADs were significantly older at diagnosis (*p* = 0.001), more hypertensive (*p* = 0.01), and displayed larger white matter hyperintensities on brain magnetic resonance imaging (MRI; *p* = 0.05). There was a significant correlation between the CSF Aβ-42 and Aβ-40 levels with interictal epileptiform discharges and delta slowing on EEGs recordings, respectively (*p* = 0.03).

**Conclusions:** Our study suggests that CSF may not serve as a surrogate marker of epilepsy in prodromal AD and cannot circumvent the operator-dependent and time-consuming interpretation of EEG recordings. In humans, AD-related epileptogenesis appears to involve the Aβ peptides but likely also additional non-amyloid factors such as small-vessel disease (i.e., white matter hyperintensities).

## Introduction

Thanks to the quantification of neurodegenerative biomarkers (i.e., Aβ peptides, p-Tau and T-Tau), cerebrospinal fluid (CSF) provides a good level of evidence for the *in vivo* diagnosis of Alzheimer's disease (AD) at the mild cognitive impairment (MCI) stage (1–3). However, it remains unknown whether the CSF can provide additional evidence of AD-related epilepsy, a frequent comorbidity affecting up to 15% of the sporadic late-onset forms of the disease (4), although it is established that seizures have an impact on the CSF neurodegenerative biomarkers (5) and that the same biomarkers are associated with changes in brain rhythms (6, 7). In daily practice, this is of clinical relevance because the diagnosis of epilepsy in prodromal AD can be very challenging: Clinical features may be subtle and/or misleading, consisting in focal seizures with non-motor features (i.e., subjective symptoms, cognitive fluctuations, or fluctuating confusional state) rather than in seizures with motor signs (whether focal or generalized), and standard electroencephalography (EEG) lacks the appropriate sensitivity (EEGs can be normal in up to 85% of epileptic AD patients) (8–10). The need for additional indicators of comorbid epilepsy in incipient AD is therefore real, and there have been substantial efforts to meet it. *First*, with neuropsychological detailed assessments: At baseline, epileptic patients display more frequent multi-domain alterations, which means that their impaired memory is commonly combined with other neuropsychological deficits such as poor visuospatial performances and/or greater functional impairment in daily living; they also show a more rapid impairment of language and/or visuospatial abilities in the first year of follow-up (11–13). *Second*, with electrophysiological techniques: EEG monitoring for at least 8 h or including sleep has been shown to be reliable with a sensitivity of 60–80% (9, 10), the coupling of EEG with magnetoencephalography can also be contributive as well as internally placed electrodes over the mesial temporal structures through the foramen ovale (14, 15). *Third*, with volumetric brain magnetic resonance imaging (MRI) protocols: Epilepsy is associated with greater atrophy in the temporal or parietal lobes (12, 16). The limitations of all the aforementioned approaches are that they require technical expertise and/or appropriate equipment, and that they are time-consuming and/or interpreter-dependent as well. By contrast, the CSF profile is relatively easy to collect and to interpret with established cut-offs (1–3). Furthermore, it offers the possibility of an *in vivo* pathophysiological study of AD-related epileptogenesis, which has been demonstrated to be amyloid driven and Tau-dependent in animal models (17, 18), suggesting tight links between the amyloid cascade and an aberrant brain hyperexcitability.

In the current study, we retrospectively compared the CSF profiles of epileptic and non-epileptic prodromal AD patients to examine the relationship of the CSF profiles with the diagnosis of prodromal AD-related epilepsy and to understand their potential pathophysiological contribution.

## Materials and Methods

### Participants

**Figure 1:**
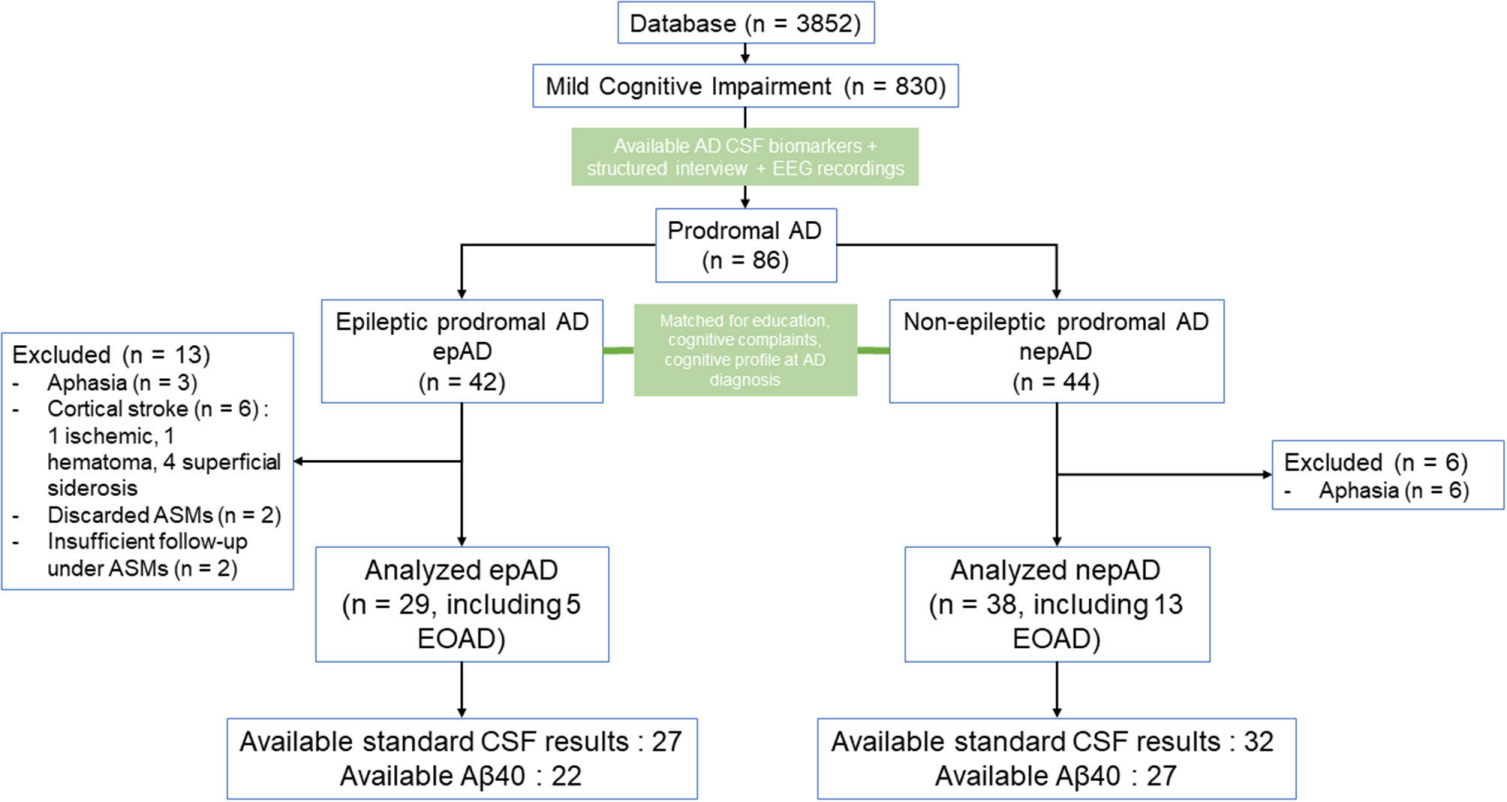
Study flow chart. ASMs, antiseizure medications; CSF, cerebrospinal fluid; EOAD, early onset Alzheimer's disease; LOAD, late onset Alzheimer's disease.

We searched the database of the Strasbourg University Hospital Memory Center (*n* = 3,852) for all patients who presented with cognitive decline between 2009 and 2017 and met the revised diagnosis criteria for MCI (*n* = 830, including 364 of the amnestic type): Since daily living activities were preserved, patients showed slight impairment (clinical dementia rating = 0–0.5) on comprehensive cognitive testing (including the evaluation of memory, praxic abilities, visual construction, executive functioning, and language) (19). Among these MCI patients, we first searched for subjects with a diagnosis of AD [based on cognitive profile with confirmative CSF biomarkers, being early onset AD (EOAD) patients when cognitive decline started before 65 years or late onset AD (LOAD) when it started at 65 years or after] (3), with available interviews [taken by the referring neurologist (F.S., F.B., or N.P.) and/or by B.C. in order to search for clinical seizures], and available EEG recordings (Figure 1). The non-epileptic patients underwent an EEG recording session as part of the diagnostic work-up for falls, transient loss of consciousness or nocturnal events that were found not to be finally epileptic in nature. The medical history was taken from patients and caregivers: We recorded the time of the first cognitive changes and, if present, the time of the first spells of unresponsiveness and/or epileptic symptoms. In epileptic patients, the age of the first EEG recording was considered as the age of suspected epilepsy without being confirmed. If interictal epileptiform discharges (IEDs) were present on EEG, epilepsy was considered as probable and was considered certain only if there was a clear-cut response to antiseizure medication (ASM) administration. The diagnosis of epilepsy was ascertained based on three main arguments: (1) recurrent stereotyped and well-demarcated transients with ictal semiology suggestive of seizures according to current guidelines (20); (2) no arguments in the medical workup for an etiology other than seizure to account for the iterative transients; (3) drug response (i.e., more than 50% improvement in the frequency of spells with ASM). All patients fulfilled the International League Against Epilepsy criteria for the clinical diagnosis of epilepsy: at least two unprovoked seizures occurring more than 24 h apart or a single seizure and a high risk (≥60% over the following 10 years) of seizure recurrence (excluding patients with provoked seizures, including cholinesterase inhibitor-induced seizures) (21). Prodromal AD was considered as causative of epilepsy because there were no other explanations in the medical workup. Epileptic prodromal AD patients (epADs) were identified and matched with consecutive non-epileptic prodromal AD control participants (nepADs) for sex, education, cognitive complaints, and cognitive profile (MCI) at diagnosis of AD (Figure 1) but not for age or Mini-Mental Status Examination (MMSE) score at diagnosis (which are influenced by underlying epilepsy in AD) (4). The exclusion criteria for all participants were a history of Korsakoff's syndrome, alcohol or substance abuse within 5 years of cognitive impairment onset, untreated B_12_ or folate deficiency, untreated conditions (hypothyroidism, Lyme disease, syphilis, HIV infection), significant medical illness (e.g., end-stage cardiac insufficiency or cancer, renal insufficiency requiring dialysis, symptomatic liver disease, respiratory condition requiring oxygen), and a pacemaker or other ferromagnetic material. The following significant neuropsychiatric conditions were also exclusion criteria (Figure 1): a diagnosis of possible or probable epilepsy without proper ASM treatment, no documentation of seizure response to ASM, severe head trauma with persistent deficits, demyelinating disease, encephalitis or meningitis, hydrocephalus, intracerebral hemorrhage, ischemic vascular dementia, clinically significant lacunar infarcts, cortical stroke (including cortical microbleed and/or hemosiderosis), aphasic and posterior atrophy variants of AD (risk of overestimation of real cognitive deficits on MMSE and cognitive testing), and medications likely to affect central nervous system functions (e.g., high doses of benzodiazepines, typical antipsychotics, tricyclics, hypnotics, and antihistamines).

### Clinical Measures

Patients were compared regarding demographics, medical history (with special attention given to risk factors for white matter hyperintensities on MRI, see Table 1), and family history of neurodegenerative disease. They all underwent physical examination and MMSE at baseline (22).

**Table 1:** Patients' clinical and radiological features.

	**EpAD** **(*n* = 29)**	**NepAD** **(*n* = 38)**	**Statistics (*p*-value)**
Female (*n*, %)	12 (41.4)	19 (50)	0.5a
Education, years (mean ± SD)	11.6 (±2.8)	11.1 (±2.5)	0.4b
Age at onset of cognitive decline, years (mean ± SD)	**69.4 (±9.9)**	64.3 (±7.2)	**0.003b**
EOAD/LOAD (*n*, %)	5 (17.2)/24 (82.8)	13 (34.2)/25 (65.8)	0.12a
Age at diagnosis of prodromal AD, years (mean ± SD)	**72.8 (±8.7)**	67.5 (±7.4)	**0.001b**
MMSE at baseline (/30)	26 (±2.4)	26.3 (±2.1)	0.5b
Family history of degenerative diseases (*n*, %)	11 (37.9)	22 (57.9)	0.1a
Hypertension (*n*, %)	**17 (58.6)**	11 (28.95)	**0.01a**
Dyslipidemia (*n*, %)	17 (58.6)	15 (39.5)	0.1a
Diabetes (*n*, %)	6 (20.7)	5 (13.2)	0.4a
Smoking (*n*, %)	12 (41.4)	17 (44.7)	0.8a
Alcohol (*n*, %)	2 (6.9)	3 (7.9)	0.9a
**Imaging features**			
Hippocampal atrophy right (mean ± SD)	1.2 (±0.8)	1.2 (±1.0)	0.8b
Hippocampal atrophy left (mean ± SD)	1.2 (±0.9)	1.3 (±0.9)	1.0b
Subcortical lacunes (*n*, %)	5 (17.2)	3 (7.9)	0.2c
Subcortical microbleeds (*n*, %)	4 (14.8)	5 (14.7)	1.0c
Fazekas score (mean ± SD)	**1.4 (±0.9)**	0.95 (0.7)	**0.05b**
ChEI at AD diagnosis (*n*, %)	0 (0.0)	0 (0.0)	–

*EOAD, early-onset Alzheimer's disease; LOAD, late-onset Alzheimer's disease; a, Chi-square test; b, Mann–Whitney test; c, Fischer's exact test; d, Student's test; ChEI, Cholinesterase inhibitors*.

*Significantly different values between epADs and nepADs appear in bold*.

### Paraclinical Measures

Standard blood tests and brain MRI were systematically performed for epADs and nepADs. On available coronal reconstructions, hippocampal atrophy was staged with the visual scale of Scheltens ranging from 0 (no atrophy) to 4 (maximal atrophy) (23). On transverse MRI sequences, white matter hyperintensities were graded according to the scale of Fazekas (24); we also recorded the presence of subcortical lacunar infarction and microbleeds. For all patients, CSF levels of Aβ-42, p-Tau, and T-tau were measured after an interval of at least 1 week from a previous overt clinical seizure (whether focal or generalized). CSF Aβ-40 was available for 49 out of 67 (73.1%) patients including 22 out of 29 (75.9%) epADs and 27 out of 38 (71%) nepADs. An underlying Alzheimer neuropathology, thus defining MCI of the AD type, was retained on CSF biomarkers showing results suggestive of amyloidosis (namely, decreased Aβ-42 < 700 ng/l or Aβ-42/Aβ-40 ratio < 0.06 in the case of high amyloid producers) plus increased p-Tau (>60 ng/l) ± T-tau (>500 ng/l) (25). Aβ42, Aβ40, T-Tau, and p-Tau were measured by sandwich enzyme-linked immunosorbent assay (ELISA) using commercially available kits (INNOTEST^®^ Fujirebio Europe, Ghent, Belgium). All assays were performed according to the manufacturer's instructions and the methodology did not change during the period in which the analyses were performed. The date of pathological CSF results was considered as the date of definite AD diagnosis. Standard CSF analysis allowed for the evaluation of CSF total proteins and albumin, and for the evaluation of neuroinflammation (i.e., white cells, intrathecal IgG and IgA, and oligoclonal bands). These data were available for 59 of 67 (88%) AD patients: 27/29 (96.4%) epADs and 32/38 (84.2%) nepADs. Patients with cognitive decline starting before the age of 50 (*n* = 4) were screened for mutations accounting for autosomal forms of familial AD (PSEN1 or PSEN2 genes, mutation or duplication of the APP gene), they were all negative.

### Electroencephalogram

Electroencephalography was performed on each patient (epADs and nepADs), with the international 10–20 system for scalp electrodes in a session of at least 30-min duration. On EEGs, epileptic foci were defined as the regions of maximum electronegativity, non-epileptiform recordings included background slowing in the theta and/or delta range, while epileptiform recordings were defined by seizures and/or IEDs (sharp waves and/or spikes and/or spike waves). No sleep-deprived EEGs were performed.

### Antiseizure Medication Treatment

All epileptic patients were prescribed antiseizure medication (ASM) treatment when the diagnosis of epilepsy was suspected on ictal semiology (with or without epileptiform EEG recordings). The choice of the drug was left to the referring physician. The clinical response to ASMs was measured on patient's and caregiver's diary at 3, 6, and 12 months and at each follow-up visit.

### Statistical Analysis

Quantitative variables are described using standard position and dispersion statistics, namely, mean, median, variance, minimum, maximum, and quantiles. Qualitative variables are described with the numbers and proportions of each category. Cumulative proportions were also calculated for variables with more than two categories. The Gaussian character of the quantitative variables was evaluated using the Shapiro–Wilk test. We used the non-parametric Mann–Whitney *U* test, or Student *t*-test as appropriate, to test differences in means (or other quantitative variables) between groups and Fischer's exact test, or the chi-square test as appropriate, for comparison of two sample proportions (%). To evaluate the diagnostic performance of a binary criterion on a dependent variable, the sensitivity, specificity, positive predictive value, and negative predictive value were estimated from the associated contingency table. For the comparison of a quantitative variable between several subgroups, a one-way ANOVA was used. The risk of the first alpha species was set at 5% for all analyses. All analyses were performed using the R software (version 3.1, R Development Core Team, 2008) *via* the GMRC Shiny Stat application of Strasbourg University Hospital (2017), except for the ANOVA performed using the Statistica Software (version 13.5.0.17, TIBCO Software, 2018).

## Results

### Clinico-Radiological Features

According to our inclusion and exclusion criteria, the study comprised 67 patients: 29 epADs (including 5 EOAD) and 38 nepADs (including 13 EOAD) (Figure 1). There were no significant differences between the two groups except for the following clinical features: EpADs were significantly more hypertensive than nepADs (*p* = 0.01), and they were older at onset of cognitive decline (69.4 vs. 64.3 years; *p* = 0.003) and at diagnosis of AD (72.8 vs. 67.5 years; *p* = 0.001). Radiologically, epADs had a significant increase in white matter hyperintensity compared with nepADs (Fazekas score = 1.4 vs. 0.9; *p* = 0.05) (Table 1).

### CSF Features

**Figure 2:**
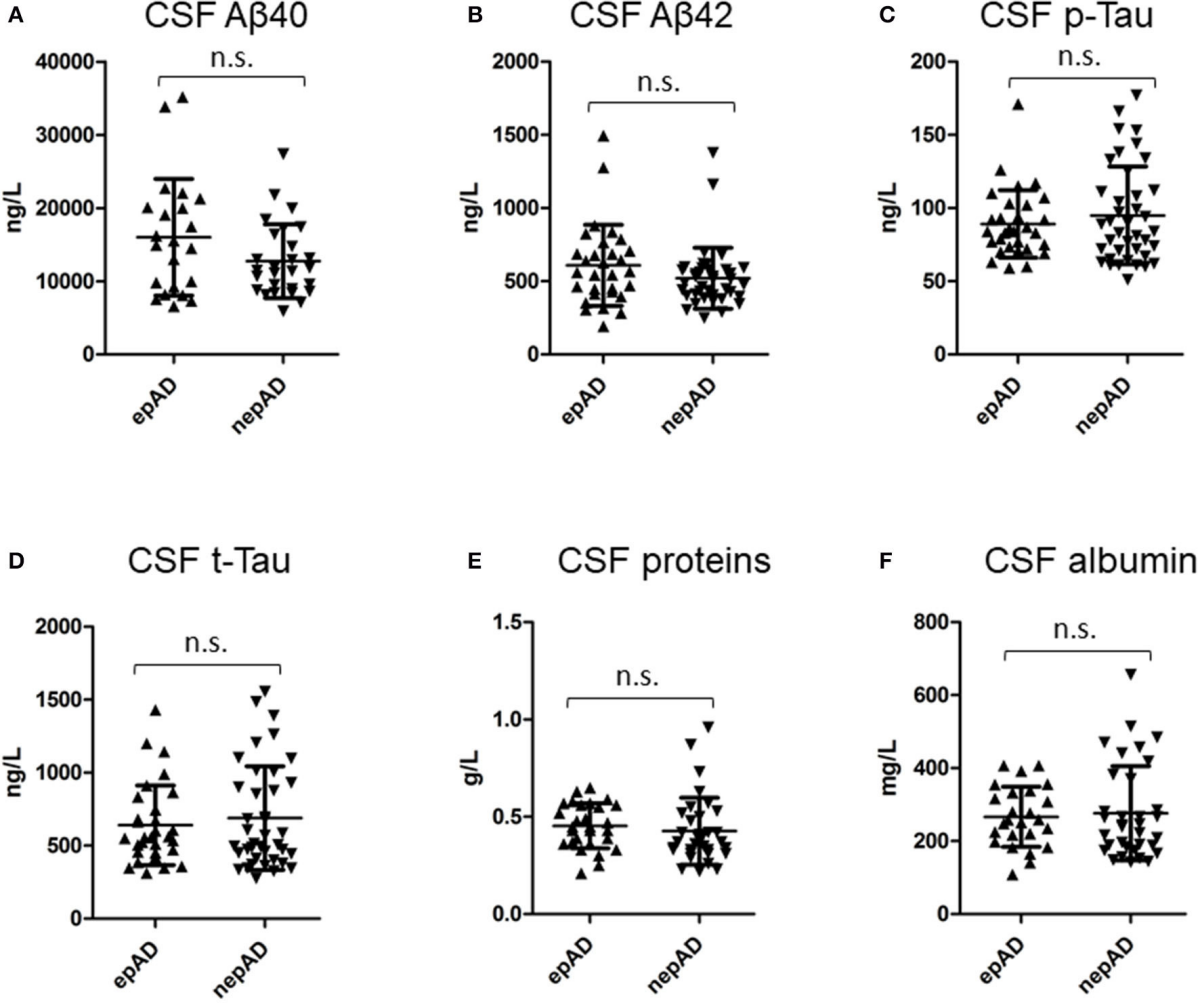
CSF compounds in epADs and nepADs.

There were no significant differences between epADs and nepADs in terms of CSF degenerative biomarkers: The CSF levels of amyloid peptides or Tau proteins and their ratios [Amyloid Tau Index (ATI), Aβ42/p-Tau and Aβ-42/Aβ-40] were not statistically different (Table 2; Figure 2). As CSF amyloid peptides are potential biomarkers of focal seizures (5), this result indicates that the overt seizure activity of epADs was well-controlled with the ongoing ASMs treatment (initiated at a mean age of 72.1 years whilst CSF was analyzed at a mean age of 72.8 years). This is in line with the known pharmacosensitivity of seizures in AD (8). But we were unable to determine any satisfactory threshold for the diagnosis of epilepsy by the use of such markers. Indeed, on a ROC curve analysis, the best discriminating value was obtained with the Aβ-40 levels: The best cut-off was set at 12,991 ng/ml (Youden index = 30.3) but it had poor diagnostic performances in differentiating epADs from nepADs, with Se = 63.6% (CI = 40.9–81.8) and Sp = 66.7% (CI = 48.15–81.5). Regarding CSF proteins, IgG, IgA, albumin, white cells, and oligoclonal bands, there were no significant differences between nepADs and epADs (Table 2).

**Table 2:** CSF features.

	**EpAD** **(*n* = 29)**	**NepAD** **(*n* = 38)**	**Statistics (*p*-value)**
**Degenerative biomarkers (mean** **±** **SD)**			
Aβ-42 (ng/l)	600 (±277)	523.5 (±210)	0.1a
P-Tau (ng/l)	90 (±23)	96 (±33)	0.9a
T-Tau (ng/l)	650 (±271)	695 (±358)	0.9a
ATI^†^ (ng/l)	0.64 (±0.35)	0.56 (±0.31)	0.2a
Aβ-42/P-Tau	7 (±3.5)	6.2 (±3.9)	0.1a
Aβ-40 (available, ng/l)	22, 16,031 (±7,966)	27, 12,751 (±5,024)	0.2a
Ratio Aβ42/ Aβ40 (available)	22, 0.05 (±0.02)	27, 0.05 (±0.02)	0.8a
**CSF standard features (mean** **±** **SD)**			
CSF proteins (available, g/l)	27, 0.45 (±0.1)	32, 0.43 (±0.2)	0.15a
CSF albumin (available, mg/l)	25, 266 (±82)	33, 277 (±131)	0.7a
CSF IgG (available, mg/l)	25, 26 (±7.5)	33, 30 (±18)	0.9a
CSF IgA (available, mg/l)	23, 4 (±1)	30, 6 (±7)	0.6a
CSF white cells (available, cells/mm^3^)	26, 2 (±2.5)	33, 1 (±1)	0.3a
Oligoclonal bands (available, *n*, %)	23, 1 (4.35)	32, 3 (9.4)	0.6c

*a, Chi-square test; b, Mann–Whitney test; c, Fischer's exact test; d, Student's test*.

† *Innotest Amyloid Tau Index*.

### Epileptological Features

**Figure 3:**
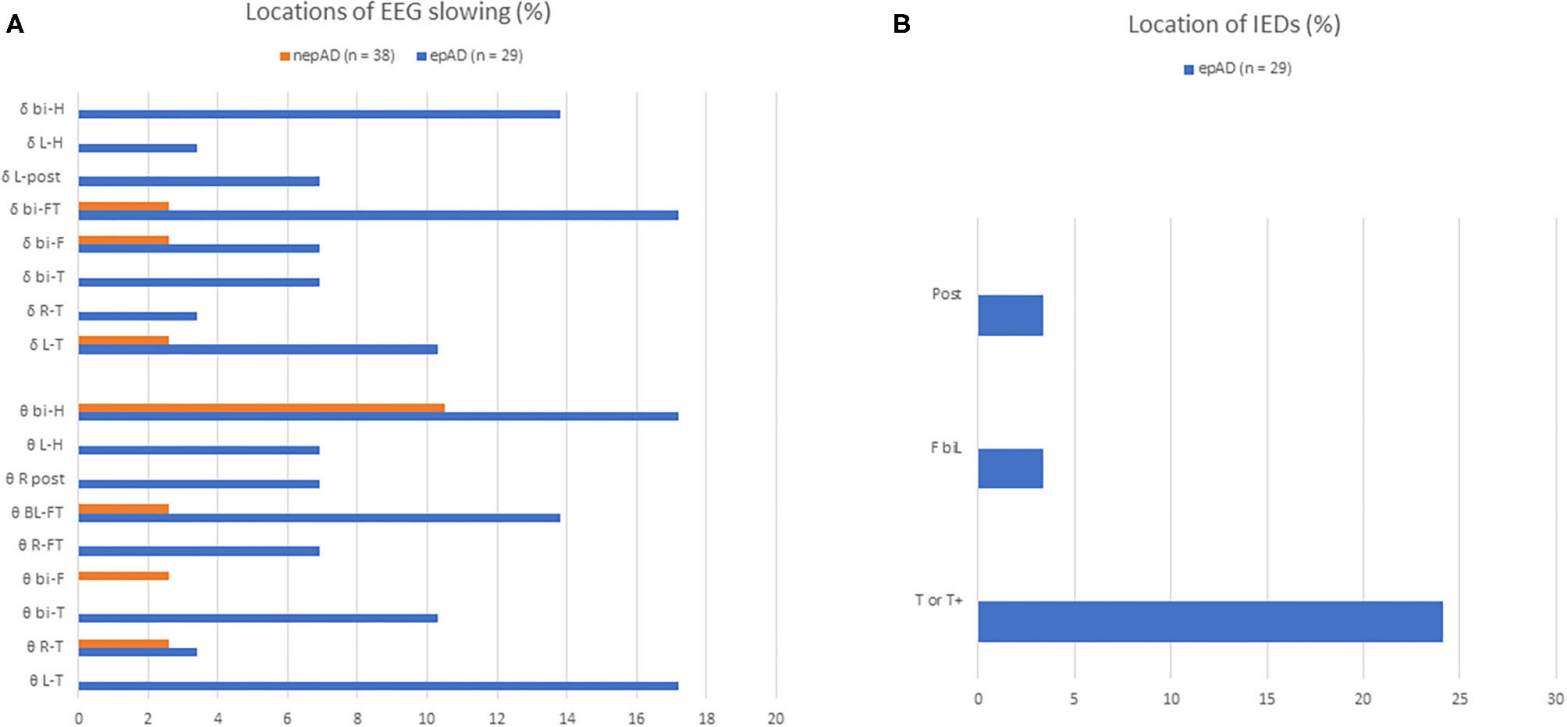
EEG features of prodromal AD patients. **(A)** Distributions of non-epileptiform activity on EEG recordings; **(B)** distributions of epileptiform activity on EEG recordings; Δ delta slowing; *θ*, theta slowing; BI-F, bi-frontal; BI-FT, bi-fronto-temporal; BI-H, bi-hemispheric; BIL-F, bilateral frontal; BI-T, bi-temporal; BI-T/T+, bitemporal or bitemporal with extension to the frontal, central or posterior electrodes; L-H, left hemispheric; L-POST, left posterior (parieto-occipital); L-T, left temporal; L-T/T+, left temporal or left temporal with extension to the frontal, central or posterior electrodes; R-FT, right fronto-temporal; R-T, right temporal; R-T/T+, right temporal or right temporal with extension to the frontal, central or posterior electrodes; R-POST, right posterior (parieto-occipital).

All epileptic patients suffered from focal seizures (Table 3), with infrequent secondary generalizations (17.2%). Focal aware seizures were less common than those with impaired awareness (27.6 and 72.4%, respectively). In the latter, the number of seizures with motor signs was twice as high as those without motor signs (Table 3). Status epilepticus was inaugural in one (3.4%) or occurred during treatment titration in two cases (6.9%). EEGs showed significantly more intermittent or continuous focal slowing in epADs than in nepADs, over various hemispheric or lobar locations (Figure 3A): 72.4 vs. 23.7%, respectively (*p* < 0.001). By contrast, seizures and/or IEDs were spatially more localized: over the temporal lobe for the former and mainly over the temporal electrodes for the latter (Figure 3B). IEDs and seizures were only recorded in the epADs group: 31 vs. 0%, respectively (*p* < 0.001). Of note, the first EEG in epADs was carried out at a mean age of 70.5 (±10.4) years, while the mean age at treatment initiation was 72.1 (±9.6) years: The recorded EEG slowing and/or epileptiform abnormalities were therefore not altered by ASMs. Nonetheless, a substantial proportion of our epADs and nepADs were treated with low-dose benzodiazepines before their first EEG (20.7 and 13.2%, respectively, *p* = 0.5), which may have normalized their recordings.

**Table 3:** Epileptological features.

	**EpAD (*n* = 29)**	**NepAD (*n* = 38)**	**Statistics (*p*-value)**
Age at seizure onset, years (mean ± SD)	67.8 (± 10.5)	NA	–
Age at treatment initiation (mean ± SD)	72.1 (9.6)	NA	–
Age at epilepsy diagnosis (mean ± SD)	72.3 (9.5)	NA	–
**Seizure semiology (** ***n*** **, %)**			
Focal seizures	29 (100)	NA	–
Focal aware^a^	8 (27.6)	NA	
Focal with impaired awareness but no motor features^b^	7 (24.1)	NA	
Focal with impaired awareness and motor signs^c^	14 (48.3)	NA	
Generalizations	5 (17.2)	NA	
Focal status epilepticus	3 (10.3)	NA	
**EEG features (** ***n*** **, %)**			
*Non-epileptiform*	**21 (72.4)**	9 (23.7)	** <0.001a**
Theta rhythms	**17 (58.6)**	8 (21.05)	** <0.001a**
Delta rhythms	**16 (55.2)**	3 (7.9)	** <0.001a**
*Epileptiform*	**9 (31)**	0 (0.0)	** <0.001c**
IEDs	**8 (27.6)**	0 (0.0)	** <0.001c**
Seizures	2 (6.9)	0 (0.0)	0.2c
EEG performed under low-dose BDZ^d^ (*n*, %)	6 (20.7)	5 (13.2)	0.5c
**Pharmacoresistant/refractory seizures (** ***n*** **, %)**	0 (0.0)	NA	–
**Seizure freedom at 6 months (** ***n*** **, %)**	18 (62)	NA	–

*BDZ, benzodiazepines; NA, not applicable; a, various auras with chest warmth, ascending aura, vertigo, paresthesia, etc.; b, possible aphasia, amnesia, confusion, agnosia, etc.; c, possible dysarthria, limb dystonia, clonic jerks, atonic phenomena/fall, automatisms (oral and/or motor); d, previously prescribed for various reasons (anxiety, insomnia, etc.). Significantly different values between epADs and nepADs appear in bold*.

### Correlation Analysis of Electrical and CSF Features of AD Patients

**Figure 4:**
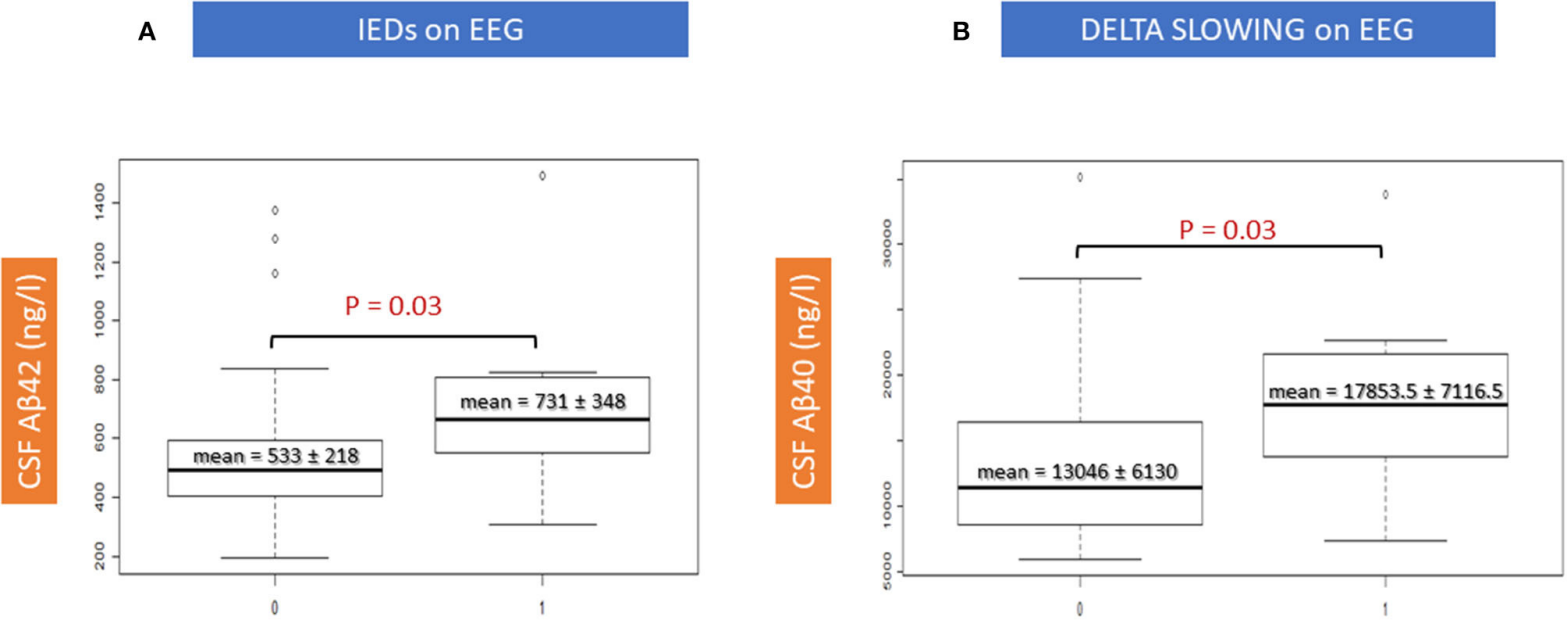
Correlations between EEG features and CSF amyloid peptides. IEDs, interictal epileptiform discharges.

There were two significant correlations between CSF amyloid features and EEG characteristics (Table 4). An increase of the mean Aβ42 levels was significantly correlated with the presence of IEDs on EEG recording and an increase of the mean Aβ40 levels was significantly correlated with the presence of EEG delta slowing (*p* = 0.03, respectively; Figures 4A,B). Moreover, an increase of the mean ATI tended to correlate with the presence of IEDs on EEG: 0.80 (±0.34) in IED patients vs. 0.57 (±0.32) in no-IED patients (*p* = 0.06). Such increase in the ratio was dependent on the increase of Aβ42 rather than on the decrease of Tau: In the IED patients vs. the no-IED patients, the mean t-Tau level was 575.4 (±148.75) ng/ml vs. 689.12 (±336.8) ng/ml, respectively (*p* = 0.3).

**Table 4:** Correlations (*p*-values) between biological and electrical features of the prodromal AD cohort (ANOVA).

	**EEG theta**	**EEG delta**	**EEG IEDs**
**CSF degenerative biomarkers**			
Aβ-42	0.3	0.07	**0.03**
Aβ-40	0.6	**0.03**	0.5
Aβ-42/Aβ-40	0.85	0.5	0.4
IATI^†^	0.6	0.1	0.06
Aβ-42/p-Tau	0.5	0.2	0.2
p-Tau	0.9	0.3	0.8
t-Tau	0.7	0.3	0.3
**CSF biochemical standard analysis**			
CSF proteins	0.9	0.6	0.7
CSF albumin	0.8	0.7	0.7

† *Innotest Amyloid Tau Index. Bold values indicate significant p value*.

## Discussion

To the best of our knowledge, this work is the first to address the potential diagnostic utility of CSF features in epileptic prodromal sporadic AD. According to our results, the CSF profile may not serve as a diagnostic tool for epilepsy in a well-defined population of prodromal AD (8). Nevertheless, higher CSF levels of Aβ-40 (from 12,991 to 35,000 ng/ml) may serve as a potential warning sign and advocate for careful clinical and EEG work-up.

Our results appear to be partly contradictory to the current literature on epilepsy in AD. *First*, our epADs patients were older than the nepADs, while the contrary has been previously published in cohorts of patients with a relatively young onset of cognitive decline despite a high cognitive reserve as reflected by high educational level (9). Thus, these previously described patients may not reflect the general population and may suffer from more severe forms of sporadic AD (i.e., the hippocampal-sparing subtype of AD) thereby manifested by earlier and faster cognitive decline and by seizures. This hypothesis is reinforced by the ages similar to our patients in a study focusing on a cohort of non-cognitively impaired epileptic elderly adults: The overall mean age of onset of epilepsy was 70 ± 6.4 and was higher in patients showing abnormal CSF Aβ42 levels (72.5 ± 7.1 years) (26). *Second*, our patients did not show the increased Aβ-42/Aβ-40 ratio described in familial forms of AD, which are particularly prone to comorbid epilepsy (27): In sporadic AD (i.e., our patients), there is probably an impaired clearance of Aβ-42 form the brain rather than an increase of its production, which might explain this observation (28). In addition, ASMs were prescribed at 72.1 years (which is more than 6 months before CSF sampling performed at a mean age 72.8 years) and may have altered the CSF biomarker profile by limiting the synaptic activity that usually promotes Aβ production and secretion (29). Another explanation is that unnoticed subclinical and/or subtle seizures may have occurred near the time of the CSF collection and thus may have altered the ratios of CSF biomarkers (5, 15). *Third*, we did not replicate the known association between EEG theta slowing and Aβ/p-Tau ratios even though the first recorded EEG was carried out on ASM-naïve patients. Still, a substantial proportion of our epADs and nepADs were treated with benzodiazepines before their first EEG, which may have normalized the recordings (Table 3). Furthermore, the published studies analyzed healthy volunteers or a combination of patients at different stages (subjective impairment, MCI, dementia) of miscellaneous degenerative diseases without associated epilepsy: This may account for such discrepancy (6, 7).

The use of CSF data at an early stage of AD (i.e., MCI stage here) theoretically promised to offer insight into the earlier epileptogenic mechanisms of the disease that cannot easily be inferred from the few autopsy studies of epileptic AD patients (i.e., pathological analysis is made in older patients, usually at the later course of the disease when AD brains likely show entangled and severe neuropathological lesions, such as intense neuronal injuries, gliosis, diffuse atrophy, and co-pathologies) (30). Indeed, our results are interesting in light of the data obtained from transgenic animal models. In these models, where animals carry mutations resulting in very high amyloid brain burden (28), the emphasis was placed on the epileptogenic role of the Aβ peptide (18). Our findings advocate for the contribution of such *amyloidocentric* pathophysiology, namely the increased Aβ40 levels correlated with the slowing of EEG background in the delta range (*p* = 0.03), and the increased Aβ42 levels with the presence of interictal epileptiform discharges (*p* = 0.03) (Table 4; Figure 4). The fact that these significant correlations were observed only with electrical changes, but not with clinical status (i.e., no differences in the MMSE scores between epADs and nepADs) nor with hippocampal atrophy on MRI, is probably related to the potential of amyloid peptides for altering brain networks excitability, most likely through a synaptic action (31, 32). They indeed enhance neuronal excitability by impairing the GABA/glutamate balance toward an increased neuronal excitation (33). The differential effect of Aβ42 and Aβ40 CSF levels on EEG features is also interesting: These peptides may impact differentially the temporal structures, as already observed in animal models and in neuropathological studies. On the one hand, Aβ40 promotes network dysfunction by the synaptopathic action of its oligomers in key structures for cognition such as the hippocampus and the parahippocampus (34–37), and by increasing intraneuronal Tau load (38). On the other hand, Aβ42 has a well-documented epileptogenic effect (18, 39) by promoting more direct neurodegeneration and plaque accumulation (40), which may lead to the primary loss of inhibitory interneurons and then induce network hyperexcitability (41). Still, interpretation should be cautious because epileptic activity was assessed primarily on a clinical basis (i.e., with overt clinical seizures, thereby overlooking subclinical epileptiform activity), because a substantial proportion of patients were treated with benzodiazepines (low doses) at the time of EEG and because epADs were receiving ASM therapy at the time of CSF sampling. Moreover, the MCI stage is quite late in the natural of history of AD (as discussed below with the limitations of our work) and our patients were prescribed ASMs after several years of untreated focal seizures (Table 3). Therefore, it is impossible to determine whether the observed correlations are a primitively pathogenic mechanism or whether they represent an adaptative response to a long-lasting activity in an epileptic network secondarily hampered by benzodiazepines and/or ASMs. In addition, given the lack of brain PET-amyloid imaging data, we cannot determine whether the increased levels of CSF Aβ42 in correlation with IEDs relate to a lower amyloid plaques burden in epADs or to an increased production of the Aβ species.

Besides, our epileptic patients were older than nepADs, they were significantly more hypertensive, and had more white matter hyperintensities measured with the Fazekas score. This suggests the contribution of a *microangiopathic* factor. In fact, brain arteriolosclerosis progresses with aging and hypertension (42), and small-vessel diseases (SVD) are an important contributor to limbic predominant and typical AD (43, 44) and to late-onset epilepsy as well (45, 46). Thus, SVD may induce the co-occurrence of both AD and epilepsy in patients with a predisposition. This *vascular hypothesis* is reinforced by the lack of significant differences in the degenerative CSF profiles between epADs and nepADs. Intriguingly, in view of the white matter hyperintensities, we did not find any CSF proteins differences (i.e., no increased CSF albumin or total proteins) as might be expected in light of the vascular hypothesis of AD (47). Yet, a CSF protein level is determined from the whole intra-axial volume and may therefore not detect a local increase of albumin level that would be relevant to focal epileptogenesis (48). We wish to emphasize that our data provide new insights into the sporadic AD-related early epileptogenesis and suggest that it is the result of an interplay between small-vessels disease and amyloidogenic mechanisms (46–48). This explains why epileptic AD patients have a worse prognosis than non-epileptic patients (i.e., a greater clinico-radiological progression): The entanglement of the intrinsic cognitive impact of the seizures and of the interictal epileptiform discharges (IEDs) with the underlying neurodegenerative process and the vascular injuries is likely to produce the observed aggravating effect (12–14). Furthermore, our results loop together epidemiological and etiological data (i.e., the vascular etiology is the most common cause of epilepsy in the elderly and it may interact with neurodegenerative contributors) (44, 46). This may be the sole explanation for the relative rarity of epilepsy in late-onset sporadic AD (up to 15%) (4) when compared with familial AD (≈50%) (27).

We are aware of the limitations of our study, which comprise the relatively small size of our cohort and the heterogeneity of the included AD patients (mixing early and late onset individuals). The retrospective and monocentric design, with missing data, limit the power of statistical analysis. The recording of EEGs in patients taking low-dose benzodiazepines may have hampered the correlations between electrical rhythms and CSF products. The inclusion of epileptic patients only displaying overt seizures that respond to ASMs as well as the lack of extensive genetic tests (i.e., ApoE, MAPT, TREM2, CALHM1, etc.) and systematic vEEG monitoring diminishes the relevance of our results regarding AD-related hyperexcitability and epileptogenesis. The CSF biomarkers used in this work and at the MCI stage of AD prompt several comments. *First*, the CSF neurodegenerative biomarkers reach a relatively early plateau shortly before or at the time of the prodromal AD diagnosis (as underlined by publications addressing the putative pathophysiological model of AD) (49), which may alter the representativeness of the respective Aβ and Tau dynamic contributions to earlier AD-related epileptogenesis (as highlighted in animal models) (18, 50). The MCI stage of AD could thus already be too late for the use of CSF biomarkers as diagnostic tools. The plateau of the CSF neurodegenerative biomarkers may indeed account for the lack of differences in CSF profile between epADs and nepADs. Moreover, the use of ASMs before lumbar puncture may have impeded seizure-related abnormalities (including activity-dependent Aβ secretion at the synaptic level) (29). *Second*, we lacked the appropriate biochemical methods to assess the CSF levels of APP, of toxic oligomeric Aβ species, and of the pro-inflammatory cytokines (i.e., intereukin-1 and−6, etc.) that may have offered insight for the understanding of AD-related epileptogenesis. Finally, the *vascular hypothesis* suggested by our results requires additional explorations that were not available because of the retrospective design used: CSF or MRI biomarkers of blood-brain barrier disruption and/or MRI-based protocols for assessing large and small vessel disease (e.g., length, radius, and tortuosity of arterioles) could provide new insight for the diagnosis of AD-related epilepsy and its pathogenesis (i.e., these factors could affect brain plasticity and network connectivity, thereby contributing to epileptogenesis and neurodegeneration). They may also show significant interactions with CSF degenerative compounds. Consequently, further studies are needed with prospective design, larger cohorts and extensive CSF analyses before ASM therapy.

## Conclusion

In conclusion, our work shows that the CSF biomarkers cannot be used as diagnostic tools for the identification of epilepsy in AD. Nevertheless, our data provide new insights into the sporadic AD-related early epileptogenesis and suggest that it is the result of an interplay between small-vessels disease and amyloidogenic mechanisms (46, 47, 51). Such complexity was recently underpinned by the review of Sen et al. (46).

## Data Availability Statement

The raw data supporting the conclusions of this article will be made available by the authors, without undue reservation.

## Ethics Statement

The studies involving human participants were reviewed and approved by Direction de la Recherche Clinique et de l'Innovation; Hôpitaux Universitaires de Strasbourg. The patients/participants provided their written informed consent to participate in this study.

## Author Contributions

BC, GH, and FS contributed to the study design. BC, GH, OB, NP, and FS contributed in the production of the main text of the manuscript. BC, NP, FB, OB, LD, and CM-H supervised data acquisition and revised the manuscript. BC, OB, and NP performed statistical analysis. All authors contributed to the article and approved the submitted version.

## Conflict of Interest

The authors declare that the research was conducted in the absence of any commercial or financial relationships that could be construed as a potential conflict of interest.

## Publisher's Note

All claims expressed in this article are solely those of the authors and do not necessarily represent those of their affiliated organizations, or those of the publisher, the editors and the reviewers. Any product that may be evaluated in this article, or claim that may be made by its manufacturer, is not guaranteed or endorsed by the publisher.
